# Special Issue “Anticancer Drug Discovery Based on Natural Products”

**DOI:** 10.3390/ijms27041675

**Published:** 2026-02-09

**Authors:** Su-Yun Lyu

**Affiliations:** 1 College of Pharmacy, Sunchon National University, Suncheon 57922, Republic of Korea; suyun@scnu.ac.kr; Tel.: +82-061-750-3759; 2 Smart Beautytech Research Institute, Sunchon National University, Suncheon 57922, Republic of Korea; 3 Research Institute of Life and Pharmaceutical Sciences, Sunchon National University, Suncheon 57922, Republic of Korea

Cancer continues to be one of the most significant global health challenges of our time. According to GLOBOCAN estimates, there were approximately 20 million new cases of cancer and 9.7 million cancer-related deaths worldwide in 2022 [1]. Approximately one in five individuals will develop cancer during their lifetime, and around one in nine men and one in twelve women will die from the disease. With demographic projections indicating that new cancer cases will reach 35 million annually by 2050, there is an urgent need for innovative therapeutic strategies [1].

Natural products have historically played a key role in cancer drug discovery, serving as invaluable sources of novel therapeutic agents [2,3]. Between 1981 and 2019, approximately 25–50% of all approved anticancer drugs were either natural products or their derivatives [4,5,6]. Notable examples such as paclitaxel, vincristine, and camptothecin underscore nature’s remarkable potential as a reservoir of bioactive compounds with unique mechanisms of action [7,8,9,10]. With this perspective, we present this Special Issue, entitled “Anticancer Drug Discovery Based on Natural Products”, which features seven contributions that explore diverse natural sources—including plant extracts, fungal proteins, and dietary compounds—and innovative delivery strategies for cancer therapy [11,12,13]. We extend our sincere gratitude to the authors, peer reviewers, and editorial team whose contributions have made this Special Issue possible.

The contributions in this Special Issue can be grouped into five major themes: nanotechnology-based drug delivery systems, plant extract screening for novel anticancer agents, immunomodulatory proteins from natural sources, drug interactions and safety considerations, and natural PARP inhibitors for targeted therapy [14,15,16].

Nanotechnology has emerged as an effective means to overcome the limitations of conventional chemotherapy, including its poor bioavailability and non-specific distribution [17,18,19]. Yeo et al. (contribution 1) developed solid lipid nanoparticles (SLNs) loaded with purpurin-18-*N*-propylimide methyl ester (P18 N PI ME), a chlorophyll-derived photosensitizer, for enhanced photodynamic therapy (PDT). The formulated SLNs exhibited particle sizes ranging from 158.59 to 248.43 nm with zeta potentials between −15.97 and −28.73 mV and showed sustained drug release over 48 h. Importantly, the nanoparticles showed no cytotoxicity in the dark but exhibited significant phototoxicity upon light irradiation in HeLa cervical cancer and A549 lung cancer cells. Among the formulations tested, the smallest-sized formulation (F4, 158.59 nm) displayed the most effective photodynamic activity, highlighting the critical role of particle size optimization in nanoparticle-based drug delivery systems for cancer therapy.

Beyond advanced delivery systems, the systematic screening of plant extracts remains a valuable approach when identifying novel anticancer compounds with unique mechanisms of action. Two studies in this Special Issue exemplify this approach. Gazizova et al. (contribution 2) investigated the anti-tumorigenic effects of sea buckthorn (*Hippophae rhamnoides*) root extracts on head and neck cancer cells. Using methanolic extraction methods combined with LC/MS and NMR analyses, the authors found that the aqueous phase of the extract reduced the viability of FaDu cancer cells while leaving non-tumorigenic human mesenchymal stem cells (hMSCs) unaffected. Flow cytometry analysis revealed that the treated cancer cells showed increased numbers in proliferative phases after 24 h, followed by elevated apoptotic cell populations after 48 h. The bioactive compounds responsible for these effects were proposed to be a mixture of 2′-hydroxyflavone isomers.

In a complementary study, Kwon et al. (contribution 3) extensively screened 200 plant extracts to identify differentiation-inducing agents for acute myeloid leukemia (AML). From the candidates tested, *Adina rubella* Hance stem extract emerged as the most potent inducer of CD11b expression in U937, THP-1, and HL-60 AML cell lines. The mechanistic studies revealed that the extract promoted differentiation through mitochondrial reactive oxygen species (mtROS) accumulation and the subsequent upregulation of p21. The UPLC-QTOF-MS analysis identified Picroside III as a key phytochemical responsible for this differentiation-inducing activity. These findings highlight the therapeutic potential of plant-derived compounds in differentiation-based treatment strategies for hematological malignancies.

In addition to small molecule phytochemicals, bioactive proteins derived from fungi and plants offer another attractive avenue for anticancer therapy. Yang et al. (contribution 4) characterized a novel fungal immunomodulatory protein, FIP-gle2, which was cloned from *Ganoderma leucocontextum*, a newly identified species from the Tibetan Plateau. The recombinant protein (rFIP-gle2) was successfully expressed in *Pichia pastoris* with a yield of 184.18 mg/L. In vitro studies demonstrated that rFIP-gle2 significantly inhibited the proliferation of B16-F10 mouse melanoma cells and induced apoptosis in a dose-dependent manner, particularly at concentrations above 1 µg/mL. At 3 µg/mL, rFIP-gle2 effectively inhibited tyrosinase activity and reduced melanin content by downregulating the microphthalmia-associated transcription factor (MITF), tyrosinase (TYR), and tyrosinase-related proteins (TRP-1 and TRP-2). RNA-seq and Western blot analyses revealed that rFIP-gle2 exerts its effects through the mitogen-activated protein kinase (MAPK) signaling pathway, with enhanced phosphorylation of JNK, ERK, and p38 proteins.

In a complementary study focusing on plant-derived lectins, Hong and Lyu (contribution 5) investigated the effects of Korean mistletoe lectin (VCA) from *Viscum album* L. var. coloratum on breast cancer cell apoptosis and macrophage polarization using both 2D and 3D co-culture models. By co-culturing MCF-7 breast cancer cells with THP-1-derived M1 or M2 macrophages in a Transwell system, the authors showed that M1 macrophages exhibit cytotoxic characteristics and can enhanc VCA-induced apoptosis in both culture models. Conversely, M2 macrophages initially displayed a protective effect by reducing apoptosis in breast cancer cells, but this effect was reversed as a result of VCA exposure. Furthermore, VCA exhibited its ability to modulate M1 and M2 polarization in the tumor microenvironment. These findings suggest that natural immunomodulatory proteins are not only able to directly target cancer cells but can also reprogram the tumor microenvironment to enhance therapeutic efficacy.

Despite the considerable therapeutic potential of natural products, their integration with conventional cancer treatments requires careful evaluation. Hoffmann et al. (contribution 6) investigated the mechanism of action and potential drug interactions of garlic extract (GE) in prostate cancer cells. The study showed that GE reduced the proliferation of LNCaP and PC3 prostate cancer cells compared to healthy PNT2 prostate epithelial cells, although contrary effects were observed in VCaP cells. Importantly, when GE was combined with standard therapies including chemotherapy (Docetaxel), androgen deprivation therapy (Enzalutamide), and Poly-(ADP-ribose)-Polymerase inhibitors (Olaparib), the efficacy of these treatments was reduced in tumor cells. This antagonistic effect was attributed to GE-induced upregulation of the metabolic enzyme CYP2C9, which is responsible for the metabolism of approximately 15–25% of all drugs. These findings underscore the importance of evaluating the potential herb–drug interactions when natural products are used as complementary therapies and highlight the need for case-by-case assessments by healthcare professionals.

Finally, the review by Qureshi et al. (contribution 7) provides a comprehensive overview of natural phytochemicals with poly ADP-ribose polymerase (PARP) inhibitory activity for colorectal cancer management. Colorectal cancer is the third most common cancer worldwide, with projections estimating that there will be 3.2 million new cases and 1.6 million deaths annually by 2040. The PARP enzyme plays a critical role in DNA damage repair, and thus its inhibition represents a viable therapeutic strategy. The review discusses several phytochemicals that demonstrate PARP’s inhibitory potential, including genistein from soybeans, ellagic acid from fruits and vegetables, naringenin from citrus fruits, quercetin, and resveratrol from grapes. Molecular docking studies revealed that ellagic acid and genistein exhibited docking scores of −10 and −10.5, respectively, comparable to synthetic PARP inhibitors such as Olaparib and Talazoparib. Given the limited bioavailability of both synthetic PARP inhibitors (Olaparib: 13%) and lipophilic phytochemicals, the authors propose using lipid–polymer hybrid nanoparticles as advanced delivery systems. This combination approach, which encapsulates both synthetic PARP inhibitors and phytochemicals in nanocarriers, offers an innovative strategy to enhance therapeutic efficacy while potentially reducing the side effects associated with conventional chemotherapy.

The studies included in this Special Issue, when considered together, underscore the considerable diversity and therapeutic potential of natural products in anticancer drug discovery. These contributions range from nanotechnology-enhanced delivery systems that overcome the limitations of conventional photosensitizers [20,21] to systematic screening approaches that identify novel differentiation-inducing agents, immunomodulatory proteins that reprogram the tumor microenvironment, and phytochemicals with PARP inhibitory activity. Together, they illustrate the varied approaches being pursued in this field.

Several important themes are apparent in these studies. Firstly, the integration of advanced drug delivery technologies, particularly nanoparticle-based systems, with natural bioactive compounds represents a valuable strategy to enhance therapeutic efficacy while minimizing off-target effects. Secondly, the tumor microenvironment, which includes tumor-associated macrophages, is being increasingly recognized as a critical target for natural product-based interventions. Thirdly, as is exemplified by the garlic extract study, the potential for herb–drug interactions requires careful evaluation when natural products are used alongside conventional therapies [22,23].

Looking to the future, continued efforts are needed to clarify the molecular mechanisms that underlie the anticancer activities of natural products, as well as advances in computational screening, synthetic biology, and nanomedicine, which will undoubtedly accelerate the journey of these compounds from the bench to the bedside [24,25,26,27,28]. We therefore hope that this Special Issue will inspire further research and collaboration in the field of natural product-based anticancer drug discovery.
